# Therapeutic benefits of factors derived from stem cells from human exfoliated deciduous teeth for radiation-induced mouse xerostomia

**DOI:** 10.1038/s41598-023-29176-w

**Published:** 2023-02-15

**Authors:** Fumiya Kano, Noboru Hashimoto, Yao Liu, Linze Xia, Takaaki Nishihara, Wakana Oki, Keita Kawarabayashi, Noriko Mizusawa, Keiko Aota, Takayoshi Sakai, Masayuki Azuma, Hideharu Hibi, Tomonori Iwasaki, Tsutomu Iwamoto, Nobuyasu Horimai, Akihito Yamamoto

**Affiliations:** 1 Department of Tissue Regeneration, Tokushima University Graduate School of Biomedical Sciences, Tokushima, 770-8504 Japan; 2 Department of Pediatric Dentistry, Tokushima University Graduate School of Biomedical Sciences, Tokushima, Japan; 3 Department of Oral Bioscience, Tokushima University Graduate School of Biomedical Sciences, Tokushima, Japan; 4 Department of Oral Medicine, Tokushima University Graduate School of Biomedical Sciences, Tokushima, Japan; 5 Department of Oral-Facial Disorders, Osaka University Graduate School of Dentistry, Osaka, Japan; 6 Department of Oral and Maxillofacial Surgery, Nagoya University Graduate School of Medicine, Nagoya, Japan; 7 Department of Pediatric Dentistry/Special Needs Dentistry, Division of Oral Health Sciences, Graduate School of Medical and Dental Sciences, Tokyo Medical and Dental University, Tokyo, Japan; 8 Yushinkai Medical Cooperation, Tokyo, Japan

**Keywords:** Oral diseases, Mesenchymal stem cells, Regeneration

## Abstract

Radiation therapy for head and neck cancers is frequently associated with adverse effects on the surrounding normal tissue. Irreversible damage to radiation-sensitive acinar cells in the salivary gland (SG) causes severe radiation-induced xerostomia (RIX). Currently, there are no effective drugs for treating RIX. We investigated the efficacy of treatment with conditioned medium derived from stem cells from human exfoliated deciduous teeth (SHED-CM) in a mouse RIX model. Intravenous administration of SHED-CM, but not fibroblast-CM (Fibro-CM), prevented radiation-induced cutaneous ulcer formation (*p* < 0.0001) and maintained SG function (*p* < 0.0001). SHED-CM treatment enhanced the expression of multiple antioxidant genes in mouse RIX and human acinar cells and strongly suppressed radiation-induced oxidative stress. The therapeutic effects of SHED-CM were abolished by the superoxide dismutase inhibitor diethyldithiocarbamate (*p* < 0.0001). Notably, quantitative liquid chromatography-tandem mass spectrometry shotgun proteomics of SHED-CM and Fibro-CM identified eight proteins activating the endogenous antioxidant system, which were more abundant in SHED-CM than in Fibro-CM (*p* < 0.0001). Neutralizing antibodies against those activators reduced antioxidant activity of SHED-CM (anti-PDGF-D;* p* = 0.0001, anti-HGF; *p* = 0.003). Our results suggest that SHED-CM may provide substantial therapeutic benefits for RIX primarily through the activation of multiple antioxidant enzyme genes in the target tissue.

## Introduction

Head and neck carcinoma is the seventh-most common cancer worldwide, accounting for 4.9% of all malignancies^1^. Currently, complete surgical excision of cancer followed by postoperative radiotherapy (RT) is a standard therapy for head and neck carcinoma; however, RT has both acute and long-term adverse effects on normal tissues^2–4^. Frequent acute side effects include taste loss, mucositis, xerostomia, dysphagia, hoarseness, erythema, and desquamation of the skin^2,3^. Late sequelae include dental caries, osteonecrosis, subcutaneous fibrosis, and trismus^3,4^. In particular, it was suggested that radiotherapy destroys dentin and enamel microstructures and promotes subsequent microbial colony formation, increasing the incidence of dental caries^4,5^. RT-induced xerostomia (RIX) is the most frequent complication in patients receiving high-dose irradiation of the salivary gland (SG). Reportedly, 64% of long-term survivors (at least 3 years after conventional RT) experience a moderate-to-severe degree of xerostomia^6^. There are no drugs to treat RIX.

Radiation damages cells through direct or indirect action. Radiation can target DNA directly, disrupting its molecular structure, or indirectly by targeting water and organic molecules, resulting in the formation of reactive oxygen species (ROS). Most radiation-induced damage reportedly results from indirect action^7^. Excessive generation of ROS, including superoxide anions (O_2_•^−^), hydrogen peroxide (H_2_O_2_), and hydroxyl radicals (OH•), causes oxidative damage^8^. Organisms protect themselves from oxidative damage by producing antioxidant enzymes such as superoxide dismutase (SOD), glutathione peroxidase (GPx), and catalase (CAT), which directly convert ROS into H_2_O and O_2_^9^, heme oxygenase-1 (HMOX1) producing antioxidant bilirubin, and NAD(P)H dehydrogenase 1, thereby suppressing the production of ROS^10,11^. Organisms also have effective antioxidant networks in which Nrf2 is protected from continual degradation by dissociating from Keap1 in the cytoplasm and is translocated into the nucleus where it activates the transcription of multiple antioxidant enzymes^12,13^. Factors that activate multiple antioxidant enzymes can be useful in the development of drugs that prevent oxidative stress-induced diseases^14,15^.

Stem cell transplantation is expected to be a new therapeutic strategy for RIX^16,17^. Previous studies have reported that the transplantation of mesenchymal stem cells (MSCs) derived from the bone marrow^18,19^, adipose tissue^20^, and peripheral blood mononuclear cells^21^ ameliorates RIX in mouse models, primarily via paracrine mechanisms. Serum-free conditioned medium (CM) from MSCs contains a broad repertoire of trophic and immunomodulatory factors^22^. It has been shown that intravenous administration of adipose MSC-CM is therapeutic against mouse RIX^23^. These previous studies demonstrated that paracrine factors from MSCs enhanced proliferation and survival activity of cells in SG; however, the mechanisms by which MSCs protect against radiation-induced SG injury remain largely elusive.

Human adult dental pulp stem cells and human exfoliated deciduous tooth stem cells (SHEDs) are dental pulp-derived MSCs characterized by strong proliferative activity^24,25^. These cells are thought to originate from the cranial neural crest, which expresses both mesenchymal and neuroectodermal stem cell markers^26^. Our previous studies have demonstrated that SHED-CM intravenously administered in various animal disease models promotes significant recovery through activating endogenous tissue-repair activities. In the present study, we examined the therapeutic effects of SHED-CM and fibroblast CM (Fibro-CM) derived from non-MSCs for comparison in the RIX mouse model and investigated the mechanism by which SHED-CM protects SG from radiation-induced injury. The null hypothesis (H_0_) for this study was that SHED-CM injection in RIX mice would not result in different therapeutic effects or anti-oxidant effects if these mice injected with DMEM or Fibro-CM. The null hypothesis was tested against the alternative hypothesis of a significant difference in outcome (H_A_).

## Results

### Intravenous injection of SHED-CM protects skin and SG from radiation

**Figure 1:**
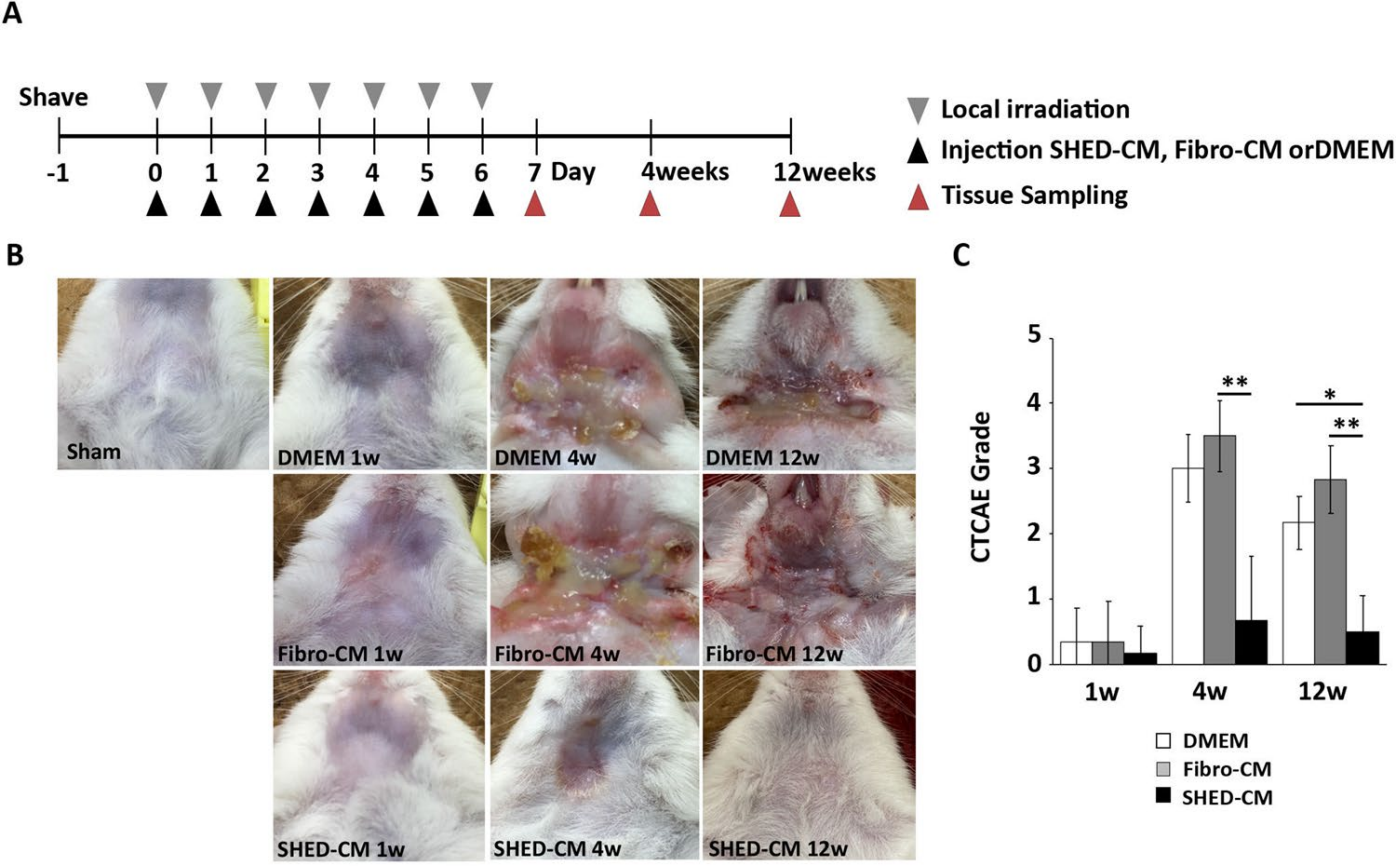
Intravenous administration of SHED-CM improves radiation-induced skin injury. (**A**) Experimental protocol. Mice were irradiated locally in the neck with 5 Gy for 7 consecutive days. SHED-CM, Fibro-CM, or DMEM was intravenously administered after each irradiation. (**B**) Representative optical images of skin injury at 24 h, 4 weeks, and 12 weeks after the first irradiation. (**C**) The level of the skin injury was evaluated based on the Common Terminology Criteria for Adverse Events scale as described in Methods. Comparisons between groups were analyzed using a Kruskal–Wallis test/Dunn's multiple comparison test (*p < 0.05, **p < 0.01). CM, conditioned medium; Fibro, fibroblast; SHED, stem cells from human exfoliated deciduous teeth.

The mice were shaved before irradiation and were locally irradiated with 5 Gy for 7 consecutive days. The irradiated mice were injected daily with DMEM, Fibro-CM, or SHED-CM (10 µL/g) into the tail vein immediately after irradiation (Fig. 1A). Four and twelve weeks after this first irradiation, mice that received DMEM or Fibro-CM exhibited a large cutaneous ulceration surrounded by severe erythema in the neck, whereas SHED-CM treatment suppressed ulcer formation (Fig. 1B).

**Figure 2:**
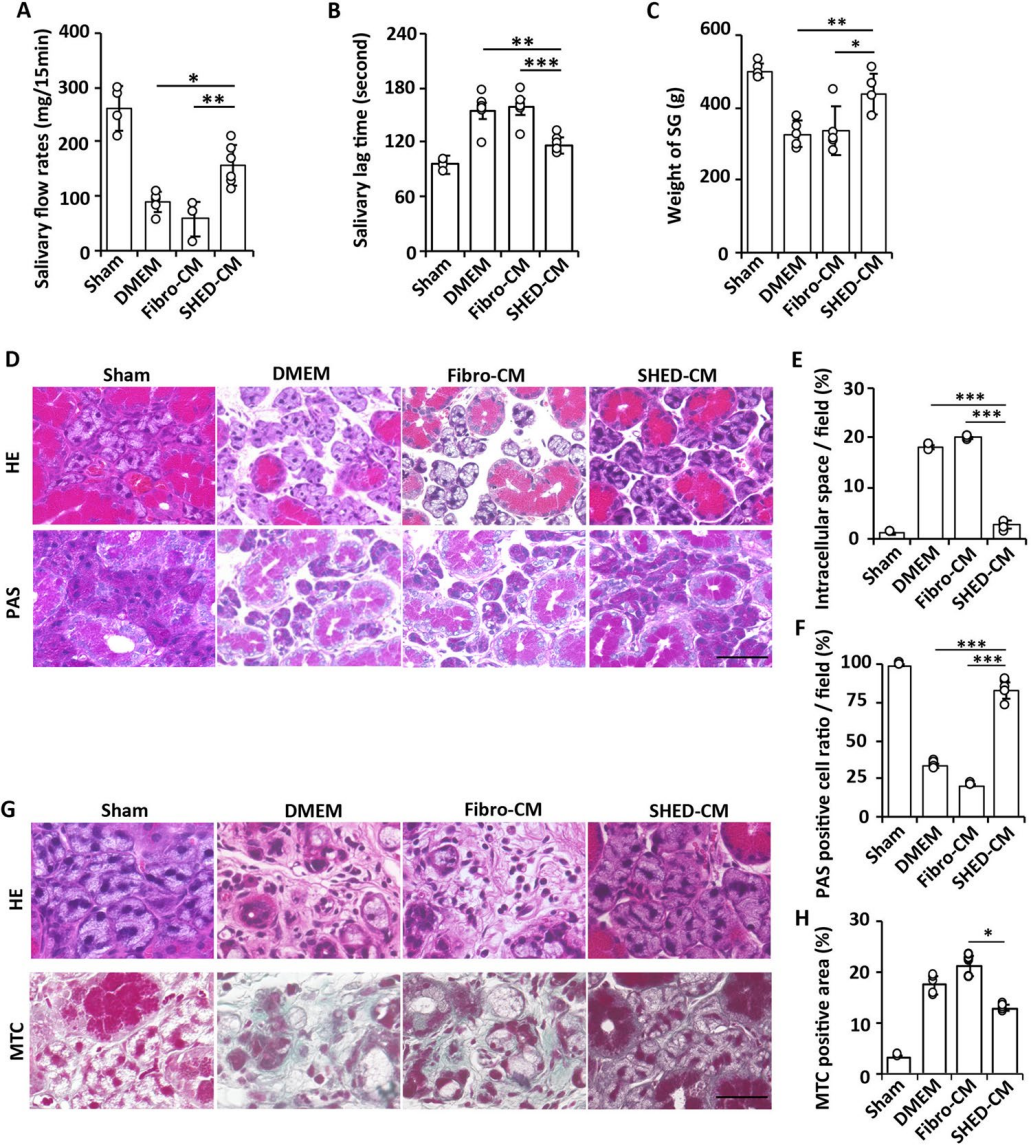
Intravenous administration of SHED-CM improves radiation-induced mouse SG injury. (**A**–**C**) Changes in salivary flow rates (**A**) and lag times (**B**) a week after the last treatment and SG weight (**C**) 12 weeks after treatment. (**D**) Images of the H&E or PAS staining of SG 24 h after the last CM treatment (n = 6). Scale bar: 100 μm. (**E**) Quantification of the intercellular space in H&E staining (the data presented by percent area per 250-μm^2^ field). (**F**) PAS-positive cell ratio in the total acinar cell number in 250-μm^2^ field. (**G**) Images of the H&E and MTC staining of SG at 12 weeks (n = 6). (**H**) Quantification of MTC-positive area (the data presented by percent area per 250-μm^2^ field). Scale bar: 100 μm. The results are expressed as mean ± SD. Comparisons between groups were analyzed using an ANOVA with Tukey's multiple comparison test [(**A**–**C**), (**E**), (**F**)], or a Kruskal–Wallis test/Dunn's multiple comparison test (H) (*p < 0.05, **p < 0.01, ***p < 0.001). CM, conditioned medium; SHED, stem cells from human exfoliated deciduous teeth. CM, conditioned medium; Fibro, fibroblast; SG, salivary gland; SHED, stem cells from human exfoliated deciduous teeth.

The evaluation based on the Common Terminology Criteria for Adverse Events (CTCAE) grade showed that the phenotypic scores of the SHED-CM group (0.67 ± 0.21 and 0.50 ± 0.22 at 4 and 12 weeks, respectively) were significantly lower than those of the DMEM (3.00 ± 0.63 and 2.17 ± 0.41 at 4 and 12 weeks, *p* = 0.093 and *p* = 0.023) or Fibro-CM (3.50 ± 0.21 and 2.83 ± 0.40 at 4 and 12 weeks, *p* = 0.009 and *p* = 0.002) group (Fig. 1C). To investigate whether SHED-CM injection improved salivary function, salivary flow rate and salivary lag time were measured at 2 weeks after the first irradiation. Mean salivary flow rate of the SHED-CM group was significantly higher than that of the DMEM (*p* = 0.014) or Fibro-CM group (*p* = 0.002) (Fig. 2A). The salivary lag time of the SHED-CM group was significantly lower than that of the DMEM (*p* = 0.001) or Fibro-CM (*p* = 0.0005) group (Fig. 2B). The weight of the SGs of the DMEM or Fibro-CM group was significantly lower than that of the sham-operated group, whereas that of the SHED-CM group was preserved (500.20 ± 18.69 mg in sham, 326.62 ± 36.59 mg in DMEM, 336.54 ± 66.19 mg in Fibro-CM, 436.60 ± 55.57 mg in SHED-CM group; SHED-CM vs. DMEM *p* = 0.008 or Fibro-CM *p* = 0.016, Fig. 2C). Histological analysis by Hematoxylin and Eosin (H&E) of SGs at day 7 revealed that the intercellular spaces of the DMEM or Fibro-CM group were expanded, possibly due to atrophy of the acinar cells; however, those in the SHED-CM group were preserved (1.20 ± 0.01% in the sham group, 18.18 ± 0.41% in DMEM group, 19.98 ± 0.32% in Fibro-CM group, 2.63 ± 0.81% in SHED-CM; SHED-CM vs. DMEM *p* < 0.0001 or Fibro-CM *p* < 0.0001, Fig. 2D and E). Similarly, PAS staining showed that the mucin-positive cell number was decreased in the DMEM or Fibro-CM group, whereas it was preserved in the SHED-CM group (99.33% ± 0.56% in sham, 33.17 ± 1.70% in DMEM, 20.33 ± 0.37% in Fibro-CM, 83.00 ± 5.97% in SHED-CM group; SHED-CM vs. DMEM *p* < 0.0001 or Fibro-CM *p* < 0.0001, Fig. 2D and F). After 12 weeks, the fibrotic area marked by Masson's trichrome staining (MTC) was increased in the DMEM and Fibro-CM groups but not in the SHED-CM group (3.28 ± 0.22% in sham, 17.61 ± 1.79% in DMEM, 21.29 ± 1.98% in Fibro-CM, 12.95 ± 0.61% in SHED-CM; SHED-CM vs. DMEM *p* = 0.614 or Fibro-CM *p* = 0.033, Fig. 2G and H).

### SHED-CM treatment preserves functional and structural marker expression in irradiated mouse SG

**Figure 3:**
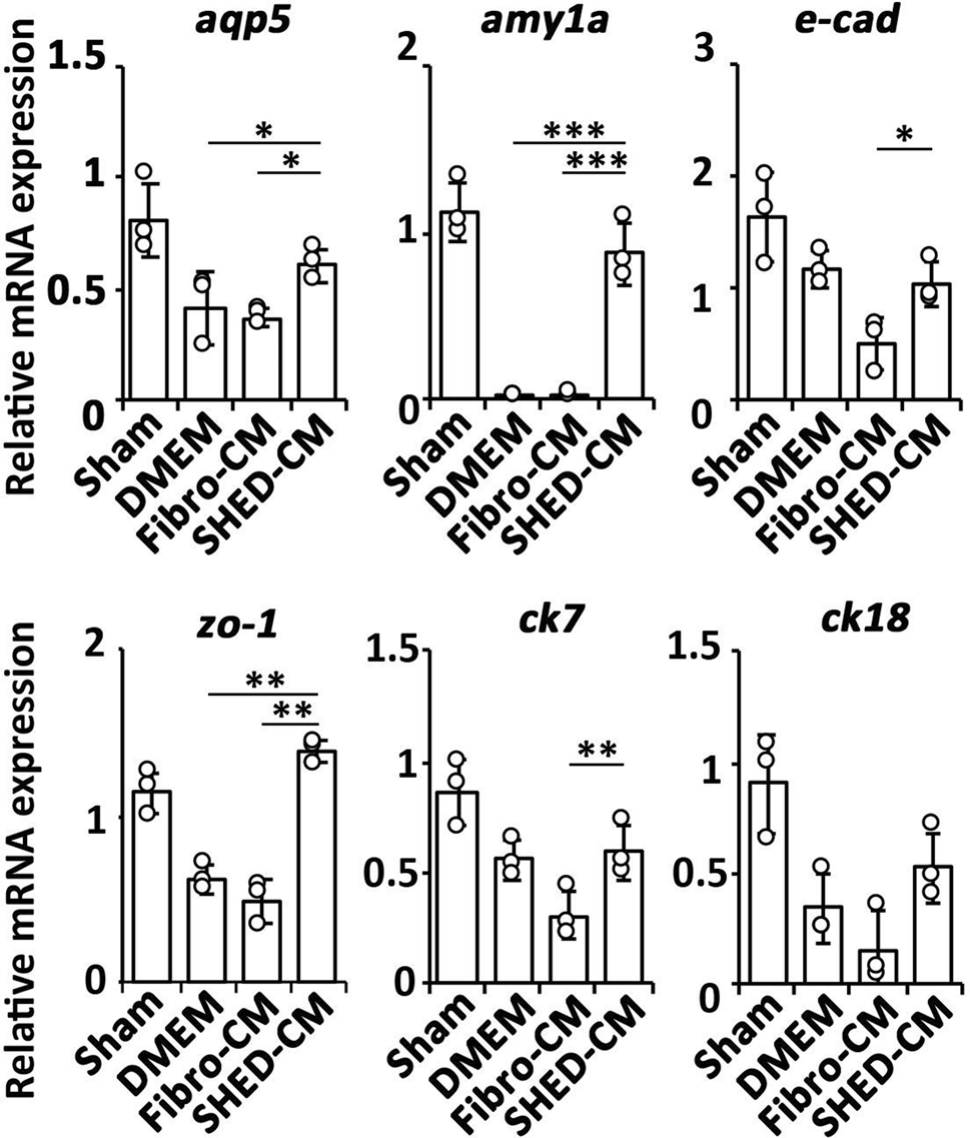
SHED-CM preserves functional and structural markers expression after mouse SG irradiation. Quanitative real-time polymerase chain reaction analysis of the indicated mRNAs in the SG 24 h after the last CM treatment. The results are expressed relative to the level in the sham group. Comparisons between groups were analyzed using an ANOVA with Tukey's multiple comparisons test (n = 3). Data are represented as mean ± SD (*p < 0.05, **p < 0.01, ***p < 0.001). CM, conditioned medium; Fibro, fibroblast; SG, salivary gland; SHED, stem cells from human exfoliated deciduous teeth.

Next, we examined gene expression of acinar and duct cell markers in irradiated mouse SGs using quantitative real-time polymerase chain reaction (qRT-PCR) analysis. The expression of acinar cell markers *aquaporin 5* (*aqp5*) and *amylase 1a* (*amy1a*), pan-epithelial markers *e-cadherin (e-cad)* and *zonula occludens-1* (*zo-1*), and the ductal markers *cytokeratin 7* (*ck7*) and *cytokeratin 18* (*ck18*) on day 7 was significantly decreased in the DMEM (vs. SHED-CM; *aqp5 p* = 0.030, *amy1a p* = 0.0001, *zo-1 p* = 0.009) and Fibro-CM (vs. SHED-CM; *aqp5 p* = 0.018, *amy1a p* = 0.0001, *e-cad p* = 0.015, *zo-1 p* < 0.0001, *ck7 p* = 0.008) groups but preserved in the SHED-CM group (Fig. 3).

### SHED-CM suppresses protein oxidation in irradiated mouse SG

**Figure 4:**
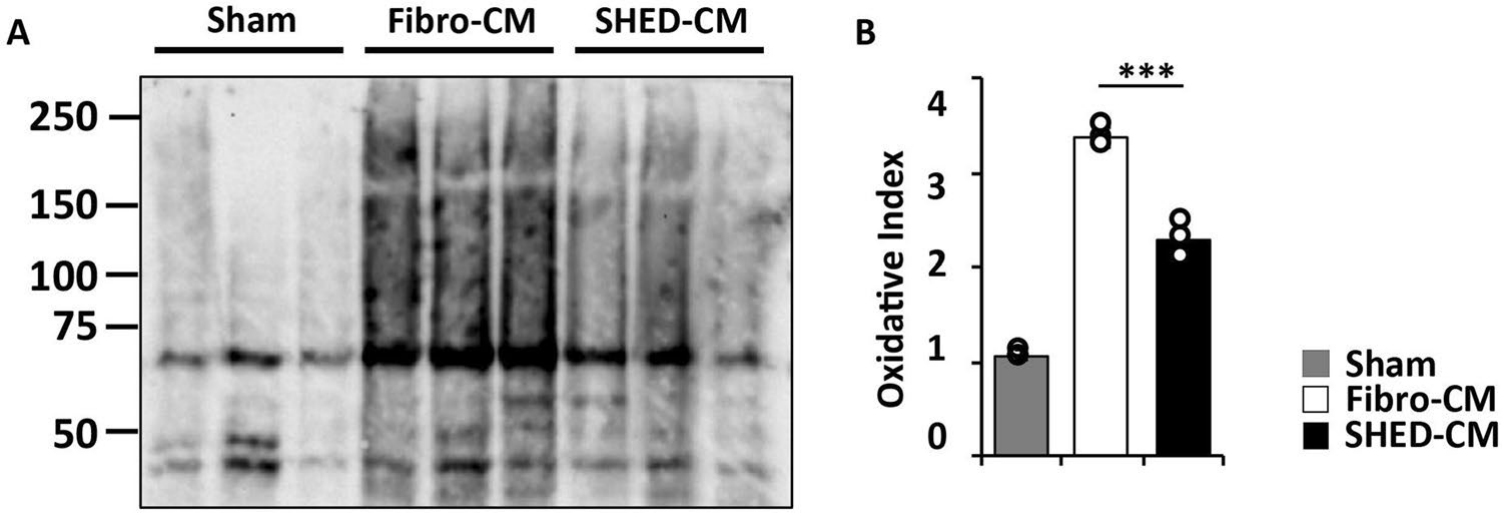
SHED-CM reduces the oxidative stress in irradiated mouse SG. (**A**) OxyBlot analysis of the irradiated SG 24 h after the last CM-treatment. (**B**) Quantitative analysis of the OxiBlot showing that SHED-CM significantly reduced the protein oxidation level compared to that in the Fibro-CM (n = 3). The results are expressed as oxidative indices, which were calculated as the relative densitometric values of the OxyBlot signal compared to those in the sham. Comparisons between groups were analyzed using an ANOVA with Tukey's multiple comparison test (***p < 0.001). CM, conditioned medium; Fibro, fibroblast; SG, salivary gland; SHED, stem cells from human exfoliated deciduous teeth.

Next, we examined the effect of SHED-CM on oxidative stress in irradiated mouse SGs using OxyBlot analysis. The level of protein oxidation in the SHED-CM group (2.16 ± 0.17) was significantly lower than that of Fibro-CM group (3.65 ± 0.20) (*p* = 0.0002, Fig. 4A and B). The full-length blot is shown in Supplemental Fig. 2.

### SHED-CM treatment increases the expression of multiple antioxidant enzymes in irradiated mouse SG

**Figure 5:**
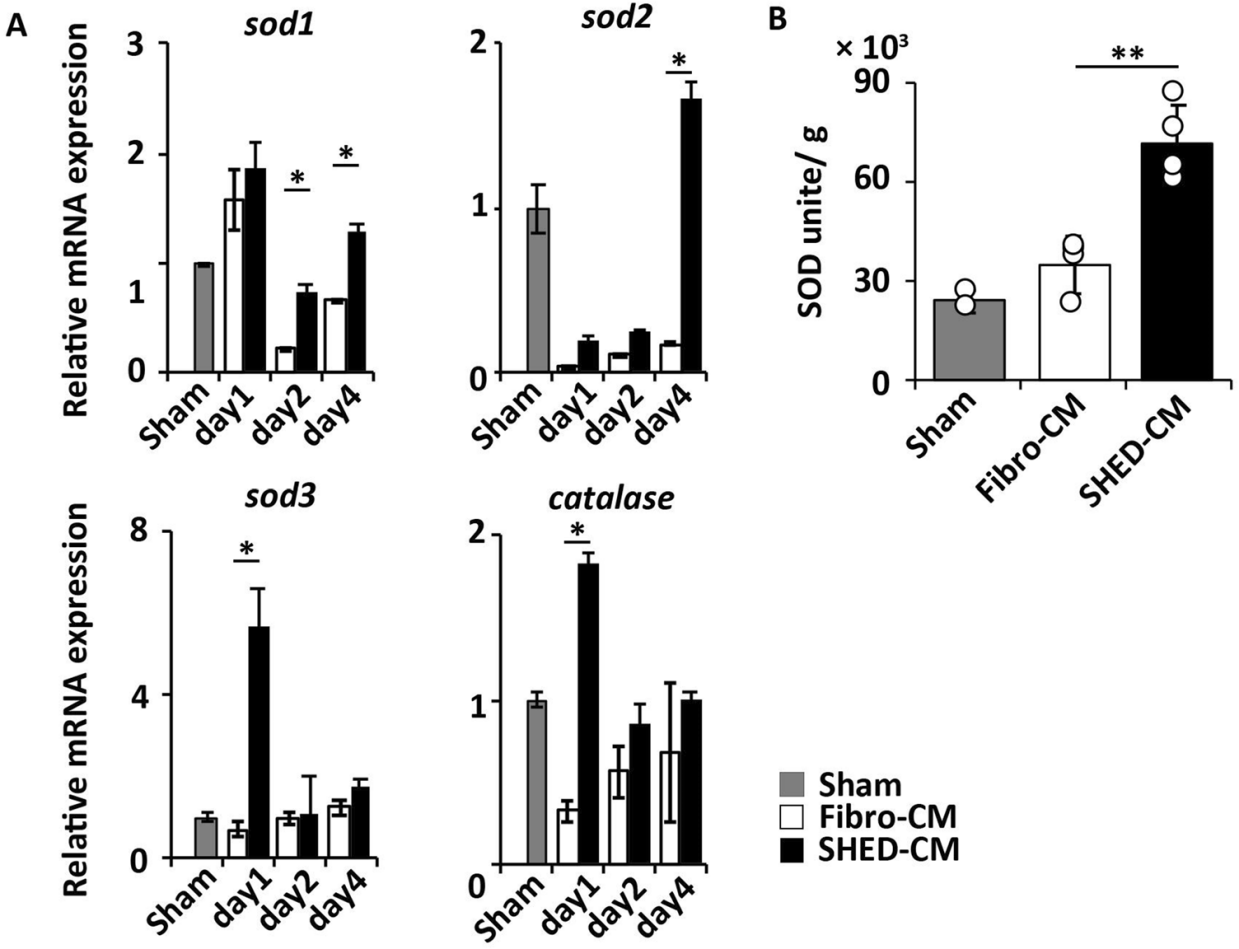
SHED-CM increases the expression of antioxidant enzymes in irradiated SGs. (**A**) Chronological quantitative real-time polymerase chain reaction analysis of indicated mRNAs in irradiated SGs treated with SHED-CM or Fibro-CM (n = 3). (**B**) The SOD activities of the irradiated SG 24 h after the last CM treatment (n = 4). Data are represented as mean ± SD. Comparisons between groups were analyzed using an unpaired two-tailed Student’s *t*-test (*p < 0.05, **p < 0.01). CM, conditioned medium; Fibro, fibroblast; SG, salivary gland; SHED, stem cells from human exfoliated deciduous teeth; SOD, superoxide dismutase.

The qRT-PCR analysis of the antioxidant enzyme genes in irradiated SGs showed that the expression levels of *sod1*, −*2*, and −*3* and *catalase* in the SHED-CM group were significantly higher than those of the Fibro-CM group (*sod1* 48 h *p* = 0.023, 96 h *p* = 0.040, *sod2* 96 h *p* = 0.019, *sod3* 24 h *p* = 0.005, *catalase* 24 h *p* = 0.002, Fig. 5A). SOD activity was significantly higher in the SHED-CM group than in the sham or Fibro-CM group (24409.9 ± 3473.3 units in sham, 35164.5 ± 8636.2 in Fibro-CM, 71534.8 ± 11813.4 in SHED-CM, SHED-CM vs. Fibro-CM *p* = 0.003, Fig. 5B).

### SHED-CM enhances cellular proliferation of irradiated human acinar cells and activates an endogenous antioxidant system

**Figure 6:**
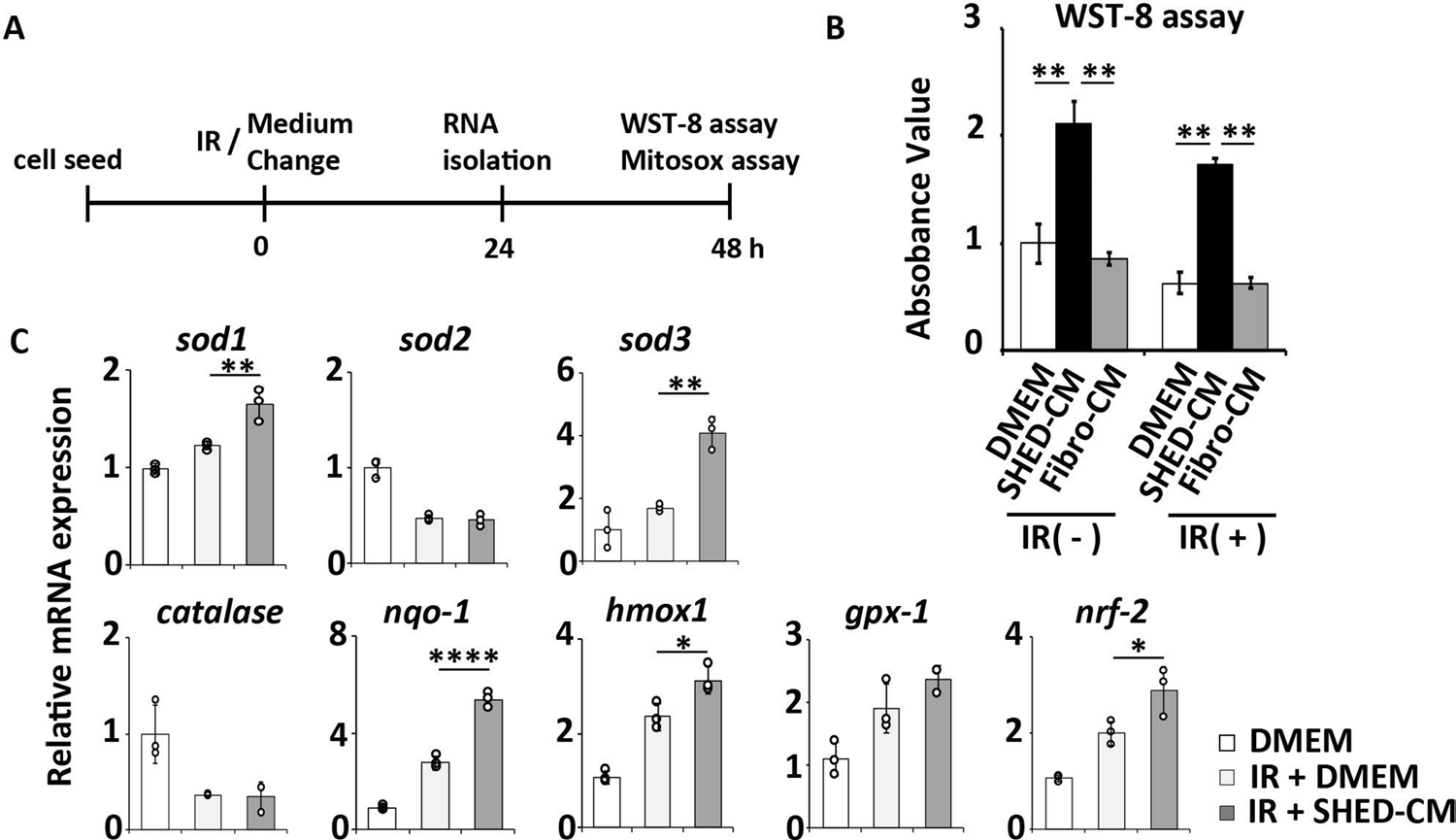
SHED-CM enhances cellular proliferation and activates the endogenous antioxidant system of the irradiated human acinar cell line. (**A**) Experimental protocol: Time course of the SHED-CM treatment in vitro. The human acinar cell line, NS-SV-AC, was irradiated with 5 Gy, and subsequently cultured with SHED-CM or Fibro-CM. (**B**) Quantitative analysis of the WST-8 assays 48 h after irradiation. (**C**) Quanitative real-time polymerase chain reaction analysis of the indicated mRNAs 24 h after irradiation. The results are expressed relative to the level in the DMEM-treated cells. Comparisons between groups were analyzed using an ANOVA with Tukey's multiple comparison test (n = 3). Data are represented as mean ± SD (*p < 0.05, **p < 0.01, ****p < 0.0001). CM, conditioned medium; Fibro, fibroblast; IR, irradiation; SHED, stem cells from human exfoliated deciduous teeth.

We examined the direct action of SHED-CM on preventing radiation-induced injury using the human acinar cell line NC-SV-AC. After irradiation, cell culture media were changed to SHED-CM, Fibro-CM, or DMEM (Fig. 6A). WST-8 analysis 48 h after CM treatment showed that the cellular proliferative activity of irradiated NS-SV-AC cells treated with SHED-CM was significantly higher than that of cells treated with DMEM (*p* = 0.012) or Fibro-CM (*p* = 0.010) (Fig. 6B). SHED-CM restored the irradiation-induced cell damage and activated the cellular proliferation machinery.

The qRT-PCR analysis showed that SHED-CM treatment significantly increased the expression of multiple antioxidant genes, including *sod1*, *sod3*, *nqo-1*, *hmox1,* and *nrf-2*, compared with DMEM (*sod1 p* = 0.005, *sod3 p* = 0.002, *nqo-1 p* < 0.0001, *hmox1 p* = 0.020, *nrf2 p* = 0.034, Fig. 6C).

### Diethyldithiocarbamate (DETCA) suppresses the antioxidative effects of SHED-CM on irradiated human aciner cells

**Figure 7:**
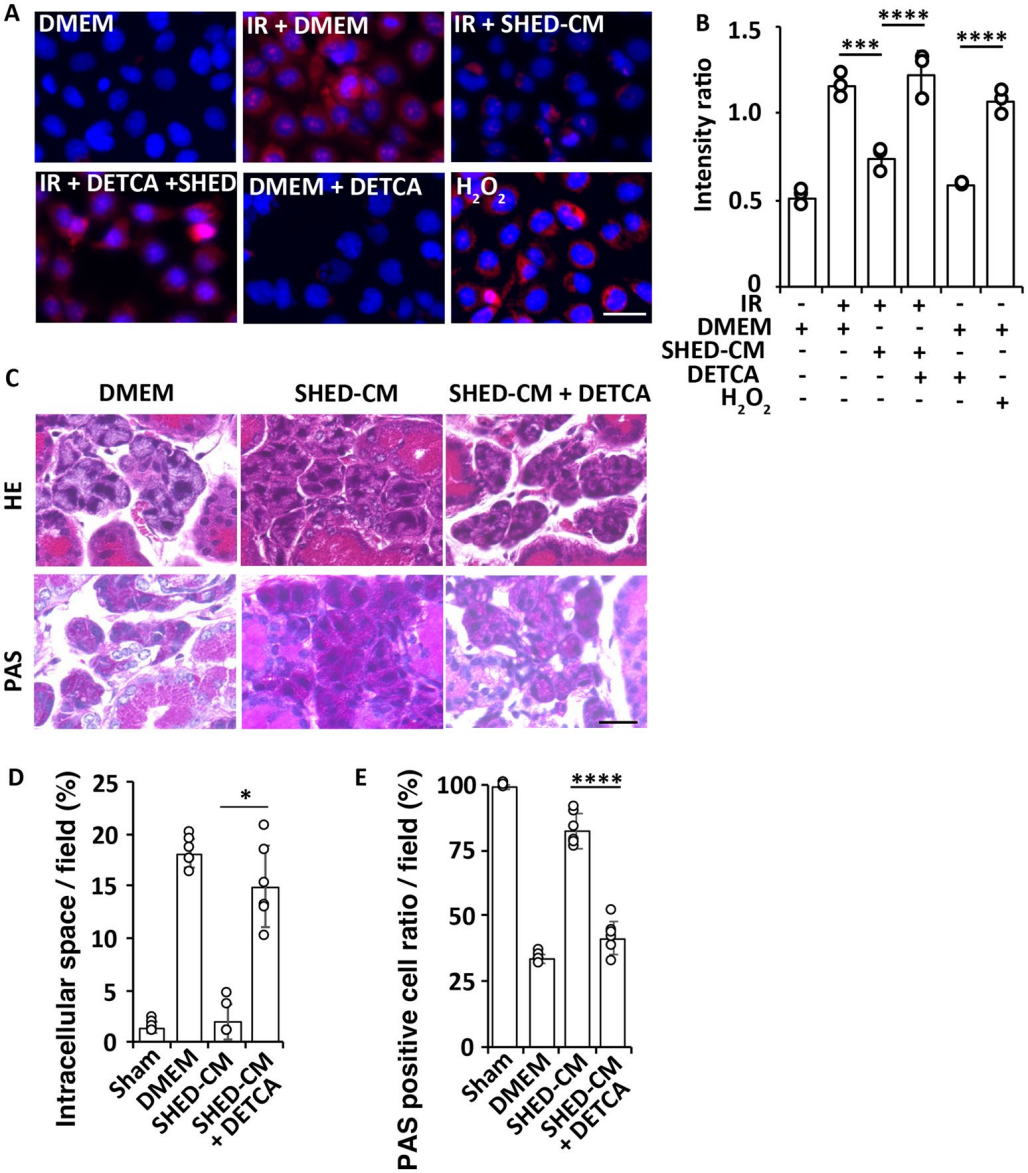
Effects of DETCA on the antioxidative and tissue preservation effects of SHED-CM after irradiation. (**A**) Representative immunofluorescence images of MitoSOX and nuclear DAPI of NS-SV-AC 48 h after irradiation. H_2_O_2_ was used as the positive control. Scale bar: 100 μm. (**B**) Quantitative analysis of the fluorescence intensities of MitoSOX analysis. The average fluorescence intensities are significantly lower in the irradiated NS-SV-AC-treated with SHED-CM than DMEM. The antioxidant effect of SHED-CM is abolished after treatment with the SOD inhibitor DETCA (n = 3). (**C**) Representative images of the H&E or PAS staining of the mouse SG at 24 h after the last CM treatment (n = 6). Scale bar: 100 μm. (**D**) Quantification of the intercellular space in the H&E staining (data presented by percent area per 250-μm^2^ field). (**E**) PAS positive cell ratio in total acinar cell number. Data are represented as means ± SDs. Comparisons between groups were analyzed using an ANOVA with Tukey's multiple comparison test [(**B**), (**E**)] or a Kruskal–Wallis test/Dunn's multiple comparison test (**D**) (*p < 0.05, **p < 0.01, ***p < 0.001, ****p < 0.0001). CM, conditioned medium; DETCA, diethyldithiocarbamate; Fibro, fibroblast; SG, salivary gland; SHED, stem cells from human exfoliated deciduous teeth; SOD, superoxide dismutase.

We examined the level of mitochondrial ROS activity in irradiated NS-SV-AC cells using MitoSOX analysis. Expression of MitoSOX increased 48 h after irradiation; however, it was significantly suppressed by SHED-CM treatment. Notably, treatment with the SOD inhibitor DETCA abolished the antioxidative activity of SHED-CM (DMEM vs. SHED-CM *p* = 0.037 and SHED-CM vs. SHED-CM + DETCA *p* < 0.0001, Fig. 7A and B).

### DETCA suppresses SHED-CM-mediated amelioration of radiation-induced SG injury

Mice were locally irradiated with 5 Gy and subsequently treated with SHED-CM alone or together with DETCA for 7 consecutive days. The intercellular spaces of irradiated SGs in the SHED-CM + DETCA group were significantly expanded (1.35 ± 0.50% in sham, 18.09 ± 1.35% in DMEM, 1.89 ± 1.64% in SHED-CM, 14.92 ± 3.86% in SHED-CM + DETCA, SHED-CM vs. SHED-CM + DETCA *p* = 0.037, Fig. 7C and D), and the PAS-positive cell ratio was significantly decreased compared with the SHED-CM group (99.17 ± 0.98% in sham, 33.17 ± 1.70% in DMEM, 82.33 ± 6.44% in SHED-CM, 41.50 ± 6.35% in SHED-CM + DETCA, SHED-CM vs. SHED-CM + DETCA *p* < 0.0001, Fig. 7C and E).

### Secretome analysis of SHED-CM

**Figure 8:**
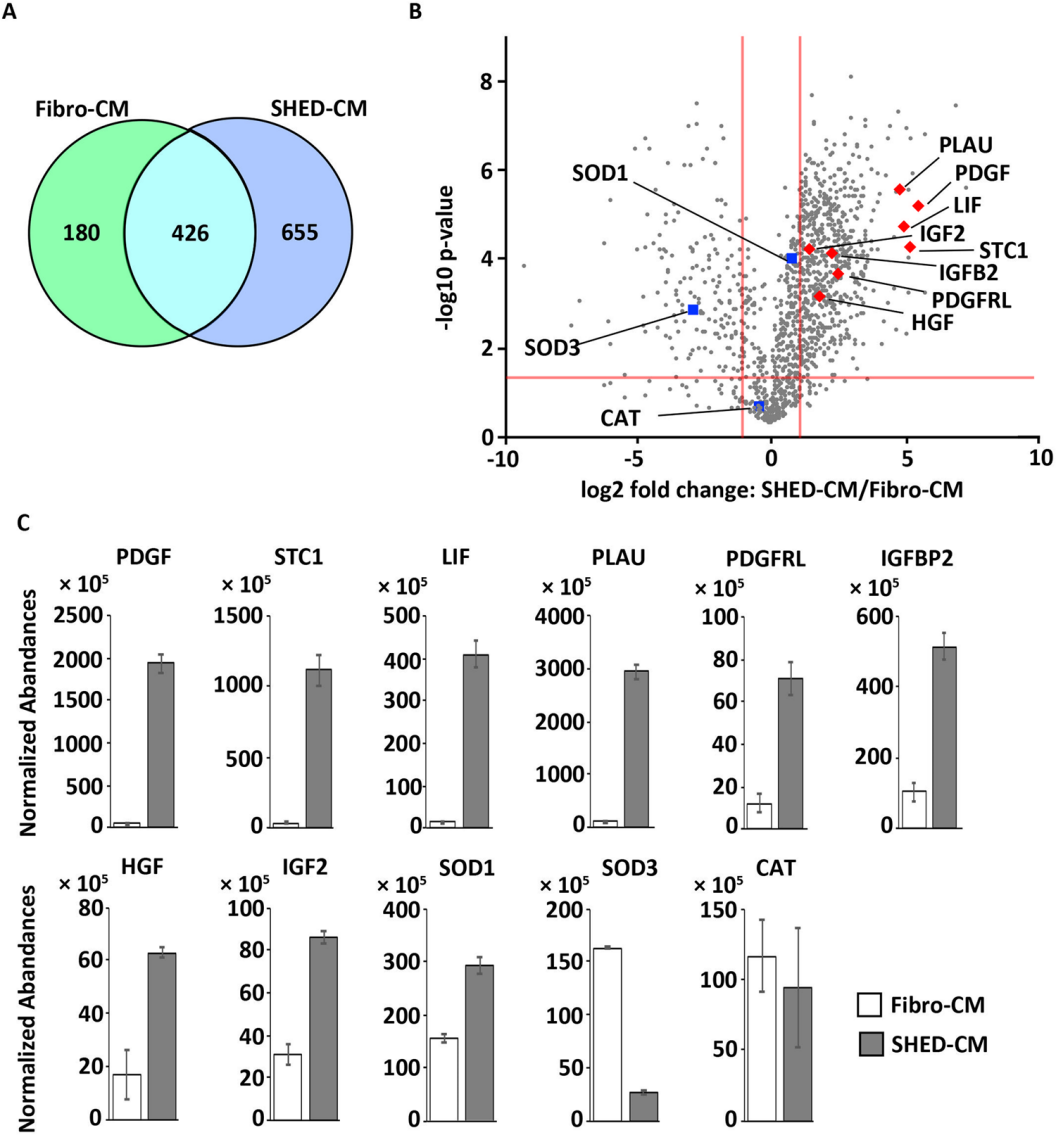
Secretome analysis of SHED-CM. (**A**) Venn diagram indicating a summary of the LC–MS/MS analysis of SHED-CM and Fibro-CM. (**B**) Volcano plot showing the differential protein profiles in SHED-CM and Fibro-CM. The *x* axis indicates the Log_2_-fold for the abundance ratio of the proteins identified in SHED-CM and Fibro-CM. The *y* axis indicates the negative log_10_ for the *p*-value of the t-test results. The non-axial vertical lines denote the ± twofold change (SHED-CM/Fibro-CM), whereas the non-axial horizontal line denotes *p* = 0.05. (**C**) An accurate amount of protein in the samples used for proteomics was measured via label-free quantitative LC–MS/MS. The normalized abundance was calculated compared with that in the Fibro-CM. The scatter plot shows the individual data, and the bar graphs indicate the mean ± SD (n = 3). CM, conditioned medium; Fibro, fibroblast; LC–MS/MS, liquid chromatography-tandem mass spectrometry; SHED, stem cells from human exfoliated deciduous teeth.

We investigated differentially abundant proteins in SHED-CM and Fibro-CM using label-free quantitative proteomics by liquid chromatography-tandem mass spectrometry (LC–MS/MS) analysis. We identified 1261 membrane-related proteins in SHED-CM and Fibro-CM, in which 655 and 180 were two-fold more abundant in SHED-CM and Fibro-CM, respectively (Fig. 8A). In SHED-CM, 625 were significantly abundant proteins, in which we identified eight proteins that have been reported to activate the endogenous antioxidant system: platelet-derived growth factor (PDGF, *p* < 0.0001), stanniocalcin 1 (STC1, *p* < 0.0001), leukemia inhibitory factor (LIF, *p* < 0.0001), urokinase-type plasminogen activator (PLAU,* p* < 0.0001), Platelet Derived Growth Factor Receptor Like (PDGFRL, p < 0.0001), insulin-like growth factor binding protein 2 (IGFBP2, *p* < 0.001), insulin-like growth factor binding protein 5 (IGFBP5, *p* < 0.001), hepatocyte growth factor (HGF, *p* = 0.001), and insulin-like growth factor II (IGF-II, *p* < 0.0001). Three antioxidant enzymes that directly scavenge free radicals were identifed: SOD3 in Fibro-CM, SOD1 and CAT in both SHED-CM and Fibro-CM (Fig. 8B and C).

### Roles of endogenous antioxidant activators in SHED-CM

**Figure 9:**
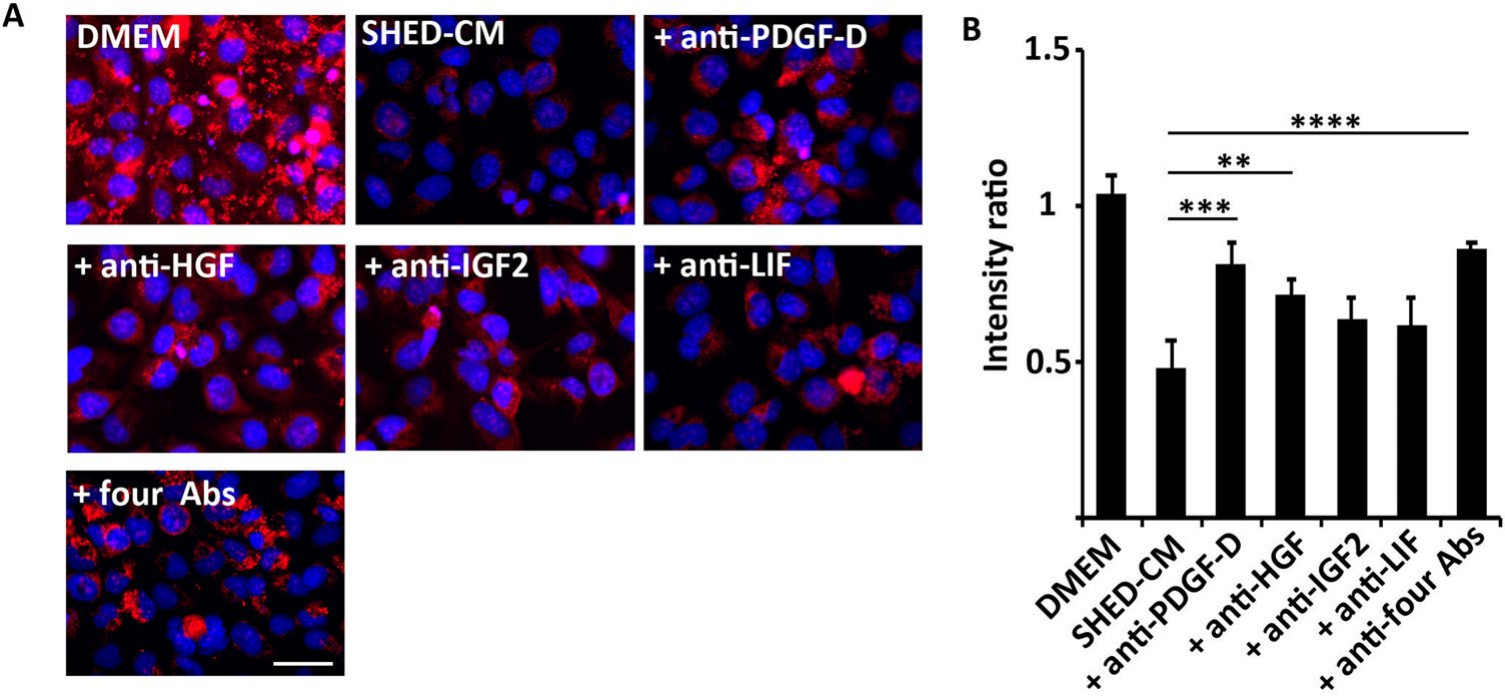
Antioxidant effect of enriched proteins in SHED-CM. (**A**) Representative immunofluorescence images of MitoSOX analysis with nuclear DAPI staining of human acinar cell line 24 h after irradiation. Scale bar: 100 μm. (**B**) Quantitative analysis of the fluorescence intensities of MitoSOX analysis. The average fluorescence intensities were significantly higher in the irradiated cells treated with SHED-CM together with anti-PDGF-D antibodies or anti-HGF antibodies than those treated with SHED-CM alone. The antioxidant effect of SHED-CM was most strongly inhibited after treatment with four neutralizing antibodies together. Comparisons between groups were analyzed using an ANOVA with Tukey's multiple comparison test. Data are represented as mean ± SD (**p < 0.01, ***p < 0.001). CM, conditioned medium; HGF, hepatocyte growth factor; Fibro, fibroblast; PDGF, platelet-derived growth factor; SHED, stem cells from human exfoliated deciduous teeth.

MitoSOX analysis with irradiated NS-SV-AC cells showed that neutralizing antibodies against PDGF-D and HGF suppressed antioxidant activity of SHED-CM (*p* = 0.0001 for SHED-CM vs. anti-PDGF-D antibody + SHED-CM, and *p* = 0.003 for SHED-CM vs. anti-HGF antibody + SHED-CM, Fig. 9A and B). Neutralizing antibodies for IGF-II and LIF also decreased the antioxidant effect of SHED-CM, although the differences were not significant (*p* = 0.051 for SHED-CM vs. anti-IGF-II antibody + SHED-CM, and *p* = 0.086 for SHED-CM vs. anti-LIF antibody + SHED-CM; Fig. 9A and B). Interestingly, treatment with four neutralizing antibodies together most strongly inhibited the antioxidant effect of SHED-CM (*p* < 0.0001 for SHED-CM vs. four antibodies + SHED-CM).

## Discussion

SHED-CM more effectively suppressed radiation-induced salivary gland damage compared to DMEM and Fibro-CM. Furthermore, SHED-CM contained multiple secreted trophic factors that activated the intrinsic antioxidant system more than Fibro-CM. Thus the null hypothesis (H_0_) was rejected.

MSC transplantation therapy holds great promise for establishing an effective treatment for radiation-induced tissue damage^27^. MSCs can self-renew, differentiate into multiple cell lineages, and secrete multiple trophic and immunomodulatory factors. It has been shown that intravenously administered MSCs accumulate in the parotid and submandibular glands and differentiate into acinar cells^18,20^. They also promote microvascularization and acinar cell proliferation and inhibit apoptotic cell death^28,29^. However, their survival rate is reportedly less than 9%, which suggests that much of the therapeutic effect of MSCs is attributable to paracrine factors from the MSCs^19^. In fact, the systemic or local administration of MSC-CM without cellular components improves the pathophysiology of RIX in animal models^23,30^. Importantly, however, the therapeutic actions of MSCs in ameliorating RIX remain largely unknown. In the present study, we analyzed the effects of SHED-CM against severe and hyperacute radiation-induced SG injury caused by continuous irradiation. We demonstrated that SHED-CM ameliorated RIX pathology and suppressed radiation-induced oxidative stress. Notably, the anti-RIX effects of SHED-CM were abolished by treatment with the SOD inhibitor DETCA, suggesting that the major anti-RIX activity of SHED-CM was exerted via inhibiting oxidative stress.

Our previous studies have shown that treatment with SHED-CM restores various types of intractable disease models due to its anti-inflammatory, M2 macrophage-inducing^31^, anti-fibrotic^32^, neuroprotective^33^, and anti-apoptotic effects^34^. The present study examined the therapeutic effects of SHED-CM on hyperacute radiation injury models, in which radiation-induced ROS play a major role in tissue injury. Therefore, this study may underestimate the anti-RIX effects of SHED-CM other than antioxidant stress. The proliferation of glandular stem cells and neovascularization supporting their nutrition would be required to restore the function of chronically damaged SG. Indeed, it has been reported that intravenous administration of SHED or SHED-CM in a mouse model of Sjögren syndrome alleviates hyposalivation^35,36^. Future studies are needed to clarify the multifaceted therapeutic effects of SHED-CM on RIX.

Irradiation-induced ROS induce cell cycle arrest and cell death^37,38^. MSCs reportedly exert antioxidant effects in various disease models, and this is considered the mechanism underlying the cytoprotective and anti-inflammatory effects of MSCs^39,40^. MSCs may enhance the antioxidant defenses of host tissues by activating expression of endogenous antioxidant enzyme genes such as *SOD*, *CAT*, *GPx*, *glutathione*, *glutamylcysteine synthetase*, *glutathione S-transferase*, *hmox1*, *nqo1*, and *bcl-2*^41^. In addition, MSCs may produce free radical scavenger proteins such as SOD, CAT, and GPx, which directly convert ROS into H_2_O and O_2_^39^. We found that SHED-CM treatment upregulated the gene expression of several antioxidant enzymes such as *sod*, *cat*, *hmox1*, and *nqo1* in both mouse SG and human acinar cell lines after irradiation. Interestingly, LC–MS/MS analysis revealed that the expression levels of SOD and CAT in SHED-CM were lower than or comparable to those in Fibro-CM (Fig. 8 and Table 1). These results suggest that the SOD and CAT in Fibro-CM were not sufficient to suppress radiation-induced oxidative stress, whereas the intrinsic antioxidant system activated by SHED-CM effectively suppressed radiation-induced oxidative stress and prevented tissue damage. The superiority of the antioxidant effect of SHED-CM over Fibro-CM was also supported by the OxyBlot data shown in Fig. 4. These analyses suggest that the antioxidant activity of SHED-CM is not due to the antioxidants produced by SHED, but rather depends on the activation of the intrinsic antioxidant system of the target cells/organs, which is induced by SHED-CM.

**Table 1:** Therapeutic factors of SHED-CM for IR-induced salivary gland injury.

	Ratio > 3.0 (vs. Fibro-CM)	References
Platelet-derived growth factor	44.75	^51^
Stanniocalcin 1	42.83	^49^
Leukemia inhibitory factor	30.26	^50^
Urokinase-type plasminogen activator	29.66	^48^
Insulin-like growth factor binding protein 2	15.76	^46,47^
Insulin-like growth factor binding protein 5	14.90	^46,47^
Hepatocyte growth factor	6.65	^44,45^
Insulin-like growth factor 2	4.54	^46,47^

CM, conditioned medium; IR, irradiation; SHED, stem cells from human exfoliated deciduous teeth.

Nrf2 is a transcription factor responsible for a variety of cellular defenses against oxidative stress. Under normal conditions, the nuclear translocation of Nrf2 is limited by proteasomal degradation; however, under oxidative stress, inhibition of degradation promotes nuclear translocation and activates the expression of various genes involved in the antioxidant defense system^13,42^. Furthermore, the nuclear translocation of Nrf2 reportedly affects the positive feedback loop of *nrf2* gene expression^43^. In the present study, the mRNA level of *nrf2* was increased in acinar cells cultured with SHED-CM. SHED-CM may increase the expression of multiple antioxidant enzymes in the acinar cells by affecting the transcriptional activity of Nrf2. However, the mechanism in which SHED-CM affects the transcriptional activity of Nrf2 remains unclear and requires clarification in future studies.

Label-free quantitative proteomics identified 625 proteins in SHED-CM at levels significantly greater than two-fold that of Fibro-CM. A cluster analysis of these proteins identified eight proteins known to activate the endogenous antioxidant system (Fig. 6 and Table 1). It was reported that HGF and bFGF secreted from human adipose-derived stem cells improve ovarian function in natural aging by reducing oxidative stress by activating the SIRT1/FOXO1 pathway^44^. HGF inhibits apoptotic cell death of cardiomyocytes by reducing oxidative stress induced by daunorubicin, serum deprivation, and hydrogen peroxide treatment^45^. IGF-II exerts neuroprotective effects by reducing oxidative stress through the acceleration of the Nrf2-nuclear translocation and by improving mitochondrial function^46,47^. Urokinase-plasminogen activator inhibits oxidative stress-induced apoptosis and DNA damage^48^. STC1 improves brain dysfunction after cerebral irradiation in rats by increasing CAT activity in the hippocampus^49^. LIF protects neurons by reducing oxidative stress during cerebral infarction by upregulating SOD3 expression^50^. PDGF protects neurons from metabolic and oxidative damage by increasing Cat, GPx, and SOD activity^51^. Despite the relatively low concentrations of activators of the endogenous antioxidant system (1–10 ng/mL) measured in SHED-CM, the combined effects of these multiple factors may provide prominent therapeutic benefits in the treatment of oxidative stress. In fact, treatment with neutralizing antibodies against PDGF, HGF, IGF-II, and LIF synergistically inhibited the antioxidant activity of SHED-CM (Fig. 9). In future, clarification of the detailed mechanisms of SHED-CM-mediated therapy of oxidative stress-related diseases or disorders will be required.

## Conclusion

We show here that intravenous administration of SHED-CM effectively prevents RIX by activating the intrinsic antioxidant stress system. This study suggests that the antioxidant activity of MSCs is not due to the antioxidants produced by MSCs but is dependent on multiple secreted trophic factors activating the intrinsic antioxidant system in target cells/tissues. The MSC-induced intrinsic antioxidant system may play a major role in the prevention of RIX.

## Methods

### Ethical compliance

Exfoliated deciduous teeth were extracted for clinical purposes from donors (aged 6–12 years) at the Yushinkai Clinic, Nagoya University, and Tokushima University Hospital. Dental pulp tissues were provided by donors who gave written informed consent. We not only confirmed donor’s intentions, but also obtained the informed consent of their parents or guardians. Participation in the study was voluntary, and no compensation was provided to the donors. This study was approved by the Institutional Ethical Committee of Nagoya University and Tokushima University Hospital and performed in accordance with the principles of the Declaration of Helsinki (Permit numbers H-73 and 3,268 for Nagoya and Tokushima University, respectively).

### Preparation of SHED-CM

SHED and SHED-CM were prepared as previously described^26^. The pulp was gently removed and digested for 1 h at 37 °C (SDminiN, Taitec, Koshigaya, Japan) in a solution containing collagenase type I (3 mg/ml) (Fujifilm Wako, Tokyo, Japan) and dispase (4 mg/ml) (Fujifilm Wako). Single-cell suspensions were plated on culture dishes in DMEM (Sigma-Aldrich, St. Louis, MO, USA), supplemented with 10% FBS (Thermo Fisher Scientific, Waltham, MA, USA), and then incubated at 37 °C in 5% CO_2_ (Forma 310 Direct-Heat CO_2_ Incubator, Thermo Fisher Scientific). The human skin fibroblast line, derived from a 36-year-old individual, was obtained at passage 12 from the Health Science Research Resources Bank (Osaka, Japan). SHED (passage 9), and Fibro (passage 13) for CM preparation were seeded at 1 × 10^5^ cells per dish. Cells at 70–80% confluence were washed with PBS and serum-free DMEM, followed by replacement with serum-free DMEM. Media were incubated for 48 h at 37 °C in a humidified atmosphere of 5% CO_2_ (Forma 310 Direct-Heat CO_2_ Incubator), then collected and centrifuged for 10 min at 2000 × g at 4 °C (AX-511, Tomy Seiko, Tokyo, Japan). Since the precipitates contained cell debris, we carefully collected the conditioned medium without nearing the precipitates. We adjusted the protein concentration of each CM to 3 µg/ml in serum-free DMEM.

### Mouse irradiation model and treatment with SHED-CM

This study was approved by the Tokushima University Animal Care and Use Committee (permission no. T2019-104). All animal experiments were performed in accordance with ARRIVE guidelines (https://arriveguidelines.org).

Male ICR mice aged 8 weeks (25–28 g) were purchased from Japan SLC (Hamamatsu, Japan) and kept in individual plastic cages and maintained at ambient temperature (22–24 °C) under a 12 h light/dark cycle. Mice were fed a standard solid diet with water ad libitum throughout the experiment and were randomly divided into one control group and three experimental groups (n = 6 per group)^52^. An overview of the experimental design and workflow is shown in Supplemental Fig. 1. Under general anesthesia with intraperitoneal injection of three types of mixed anesthetic agents: 0.75 mg/kg medetomidine (Nippon Zenyaku Kogyo, Koriyama, Japan), 2 mg/kg midazolam (Sandoz, Tokyo, Japan), and 2.5 mg/kg butorphanol (Meiji Seika Pharma, Tokyo, Japan). Necks were locally irradiated with 5 Gy for 7 consecutive days at a rate of 4 Gy/min and at a distance of 150 mm, using an MBR (Hitachi Medical, Kashiwa, Japan). Before irradiation, the mice were shaved and protected by a 2-mm filter of lead, except for the irradiated area^53^. The mice were housed individually to prevent gnawing of ulcers and other potentially damaging interactions. The irradiated mice were injected daily with SHED-CM, Fibro-CM, or DMEM (10 µL/g) into the tail vein immediately after irradiation.

### Measurement of the dermatitis score and ulcer area

Radiation-induced skin reactions were scored using CTCAE version 5.0 (https://ctep.cancer.gov/protocolDevelopment/electronic_applications/ctc.htm), published by the National Institutes of Health on November 27, 2017. Skin damage was classified on a scale of 1–5 based on the CTCAE: grade 1, faint edema with dry desquamation; grade 2, edema with restricted moist desquamation in the skin fold; grade 3, extensive moist desquamation; grade 4, skin necrosis or ulceration of full-thickness dermis with spontaneous bleeding; and grade 5, death by radiation.

### Salivary fluid secretion test

Salivary flow rates and salivary lag times were measured as described^54^. At two weeks post-irradiation, saliva was collected for 15 min after stimulation by intraperitoneal injection of pilocarpine (2 mg/kg, 161-07201, Fujifilm Wako) in PBS, and salivary flow rates were calculated. The lag time was defined as the time from stimulation to the beginning of saliva secretion.

### Histology and immunohistochemical analysis

The mice were sacrificed by intraperitoneal injection by pentobarbital injection 24 h after the last CM treatment, and the left and right SG were harvested and their weight measured. The collected SGs were used for tissue analysis, total RNA isolation, and SOD assays. SGs were fixed in 4% paraformaldehyde (02890-45; Nacalai Tesque, Kyoto, Japan) in PBS at 4 °C for 24 h and embedded in paraffin (Sakura Finetek Japan, Tokyo, Japan), and 5-μm sections were obtained using an HM 450 Sliding Microtome (Thermo Fisher Scientific). Sections were deparaffinized and hydrated by immersion in a series of xylene (5 min × 3 times permeation) (244-00081, Fujifilm Wako) and graded alcohol solutions (5 min at each concentration: 100% × 2 times, 90%, 80% and 70% alcohol solutions, Japan Alcohol Trading, Tokyo, Japan). The sections were stained with hematoxylin and eosin (H&E) (Sakura finite), periodic acid-Schiff (PAS) (Scy Tek Laboratories, Logan, UT, USA)^23^, or Masson–Goldner trichrome (MTC) (Sigma-Aldrich), and photographed using a BZ-9000 (Keyence, Osaka, Japan). The intercellular space and PAS-positive acinar cell ratios were measured using ImageJ software (National Institutes of Health, Bethesda, MD, USA). Five different fields within the SG were randomly selected from each section and measured.

### The qRT-PCR analysis

Total RNA was isolated from 100 mg of SG by isogen II (Nippon Gene, Tokyo, Japan) according to the manufacturer's instructions. Total RNA was quantified using a spectrophotometer, and RNA integrity was checked on 1% agarose gels. Reverse transcription reactions were performed with Superscript III reverse transcriptase (Invitrogen, Carlsbad, CA, USA) using 0.5 μg of total RNA in a 20-μL total reaction volume. The qRT-PCR was performed using the Thunderbird Sybr qPCR Mix (Toyobo, Osaka, Japan) and the StepOnePlus Real-Time PCR System (Applied Biosystems, Foster City, CA, USA)^55^. Primers were designed using Primer3 (http://primer3.ut.ee, Supplemental Table1).

### Cell culture, WST-8, and MitoSOX analysis

The characteristics of the cell line NS-SV-AC (immortalized human SG acinar cells) are described in detail in the literature^56^. The clone was cultured at 37 °C in serum-free keratinocyte medium (Gibco Laboratories, Gaithersburg, MD, USA) in an incubator with an atmosphere containing 5% CO_2_ (Forma 310 Direct-Heat CO_2_ Incubator). Mitochondrial superoxide was detected using a fluorescent MitoSOX probe (Invitrogen). Cells were incubated in Hank’s buffer with 2 μM MitoSOX-red for 30 min at 37 °C in a 5% CO_2_ incubator (Forma 310 Direct-Heat CO_2_ Incubator) atmosphere and washed with PBS. For neutralization, 10 μg/ml anti-human PDGF-D antibody (MAB1159; R&D Systems), 1 μg/ml anti-human HGF antibody (MAB294; R&D Systems), 30 μg/ml anti-human IGF-II antibody (MAB292; R&D Systems), or 1 μg/ml anti-human LIF antibody (MAB250; R&D Systems) was used. The SHED-CM was incubated overnight with antibodies at 4 °C before the assay. The cells were irradiated and immediately cultured for 24 h in serum-free DMEM, SHED-CM, or Fibro-CM with or without neutralization antibodies. For quantitative analysis of cell proliferation, WST-8 solution (Cell Counting Kit-8; Dojindo, Kumamoto, Japan) was added to each well. After incubation at 37 °C for 1 h in a humidified CO_2_ incubator (Forma 310 Direct-Heat CO_2_ Incubator), absorbance at 450 nm was measured using a microplate reader (Infinite 200 PRO, Tecan Japan, Kawasaki, Japan).

### OxyBlot analysis

OxyBlot analysis was performed according to the manufacturer’s specifications (S7150; Millipore, Billerica, MA, USA). Briefly, SGs were collected and lysed in RIPA buffer (50 mM Tris–HCl, pH 7.4; 1% v/v NP-40; 1% sodium deoxycholate; 0.1% SDS; 150 mM NaCl; and 50 mM Tris–HCl) (Fujifilm Wako) and 1 mM phenylmethylsulfonyl fluoride (Fujifilm Wako). They were sonicated for 10 s and centrifuged at 14,700 × g for 30 min at 4 °C (AX-511). After centrifugation, the supernatants were collected. Protein concentrations were determined using Bradford Ultra (Novexin, Cambridge, United Kingdom). Protein samples were then mixed with the same volume of 12% SDS and incubated with an equal volume of the 1 × dinitrophenylhydrazine derivatization solution at room temperature for 15 min before reaction termination upon addition of the neutralization solution^57^. Non-reduced samples were electrophoresed on 10% TGX gel (Bio-Rad, Berkeley, CA, USA) and transferred to a PVDF membrane (Millipore). The PVDF membrane was immunoblotted with 2,4-dinitrophenylhydrazone rabbit antibody (dilution 1:150) and secondary anti-rabbit IgG HRP antibody (dilution 1:300) included in the kit. The PVDF membrane was reacted with Immobilon Forte Western HRP substrate, and the ELC signal was detected using a Chemidoc imaging system (Bio-Rad). Quantification of oxidized proteins was performed using Image Lab (Bio-Rad).

### Superoxide anion scavenging activity

SOD activity of the irradiated SGs treated with SHED-CM was measured using the SOD assay kit WST (Dojindo Molecular Technologies, Gaithersburg, MD, USA) according to the manufacturer’s instructions. The absorbance at 450 nm was meseared by plate reader (InfiniteM200PRO, Tecan Japan, Kawasaki, Japan). The IC50 was determined as the concentration of sample that inhibited the formation of WST-1 formazan by 50%. IC50 percentages were given as the mean value of three experiments.

### SOD inhibition analysis

We used DETCA (Sigma-Aldrich) to suppress SOD activity in vivo and in vitro. For in vitro and in vivo experiments, DETCA at 1 mM and 300 mg/kg per day was used, respectively, as these were the highest doses that did not show side effects. SHED-CM + DETCA (n = 6 per group) or SHED-CM (n = 6 per group) was administered daily.

### LC–MS/MS analysis

Secretome analysis of CM was performed based on a previously reported protocol^58^. In brief, SHED-CM and Fibro-CM were concentrated using an Amicon Ultra 3 K filter (Millipore). Methanol/chloroform precipitation was performed for protein purification. Resulting pellets were resolved by MS buffer (8 M urea and 50 mM Tris–HCl, pH 8.0), followed by reduction in 5 mM DTT (Fujifilm Wako) and alkylation in 27.5 mM iodoacetamide (Fujifilm Wako) for 30 min in the dark at room temperature. After being diluted eight times in 50 mM Tris–HCl (pH 8.0), proteins were digested overnight with 50 ng of Lys-C (Fujifilm Wako) and trypsin (Promega, Madison, WI, USA) at 37 °C. Peptides were purified using GL-Tips SDB (GL Science, Tokyo, Japan) based on the manufacturer's protocol. The concentration of peptides was measured using a Pierce quantitative colorimetric peptide assay kit (Thermo Fisher Scientific). Subsequently, peptides (268 ng each) were injected into an EASY-nL connected to a Q-Exactive PLUS (Thermo Fisher Scientific) mass spectrometry system. Protein identification and label-free quantification were performed using Proteome Discoverer 2.2 (Thermo Fisher Scientific).

### Statistical analysis

All data were presented as mean ± standard deviation (SD) . An analysis was performed with Graph Pad Prism (ver.9.4.1 , La Jolla, CA, USA). The distribution of the data was evaluated using the Shapiro–Wilk normality test. An unpaired two-tailed Student’s *t*-test was used to compare two groups. To analyze three or more independent groups, we used repeated measures ANOVA with the Tukey's multiple comparison test or Kruskal–Wallis test/Dunn's multiple comparison test.

## Supplementary Information

Supplementary Information.

## Data Availability

The LC-MS/MS raw data and analysis files have been deposited in the ProteomeXchange Consortium (http://proteomecentral.proteomexchange.org) via the jPOST partner repository (https://jpostdb.org) with the data set identifier PXD032142. The other datasets generated or analyzed during this study are available from the corresponding author on reasonable request.
