# Supplementary material for: Therapeutic benefits of factors derived from stem cells from human exfoliated deciduous teeth for radiation-induced mouse xerostomia
Source: Sci Rep. 2023 Feb 15;13:2706. doi: 10.1038/s41598-023-29176-w (PMC9932159; doi:10.1038/s41598-023-29176-w)
Supplement: Supplementary file 1 — Supplementary Information. [file 41598_2023_29176_MOESM1_ESM.docx]

**Therapeutic benefits of factors derived from stem cells from human exfoliated deciduous teeth for radiation-induced mouse xerostomia**

Fumiya Kano^1^, Noboru Hashimoto^1^, Yao Liu^1^, Linze Xia^1^, Takaaki Nishihara^1^, Wakana Oki^1^, Keita Kawarabayashi^2^, Noriko Mizusawa^3^, Keiko Aota^4^, Takayoshi Sakai^5^, Masayuki Azuma^4^, Hideharu Hibi^6^, Tomonori Iwasaki^2^, Tsutomu Iwamoto^7^, Nobuyasu Horimai^8^, Akihito Yamamoto^1^*


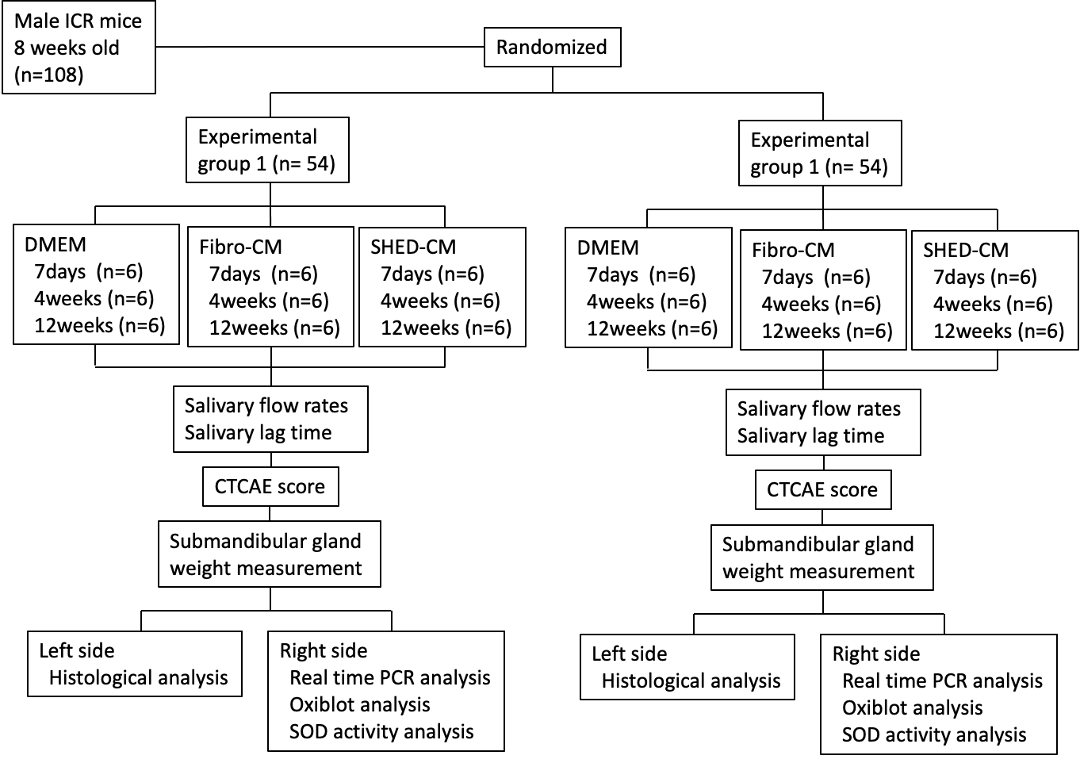
(A)


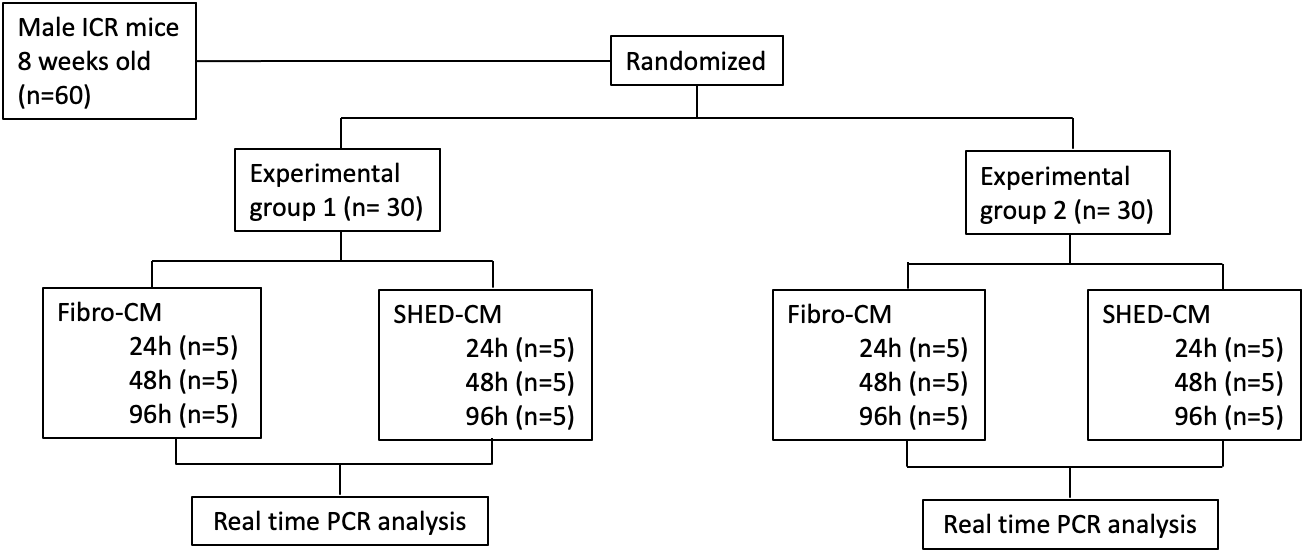
(B)

**
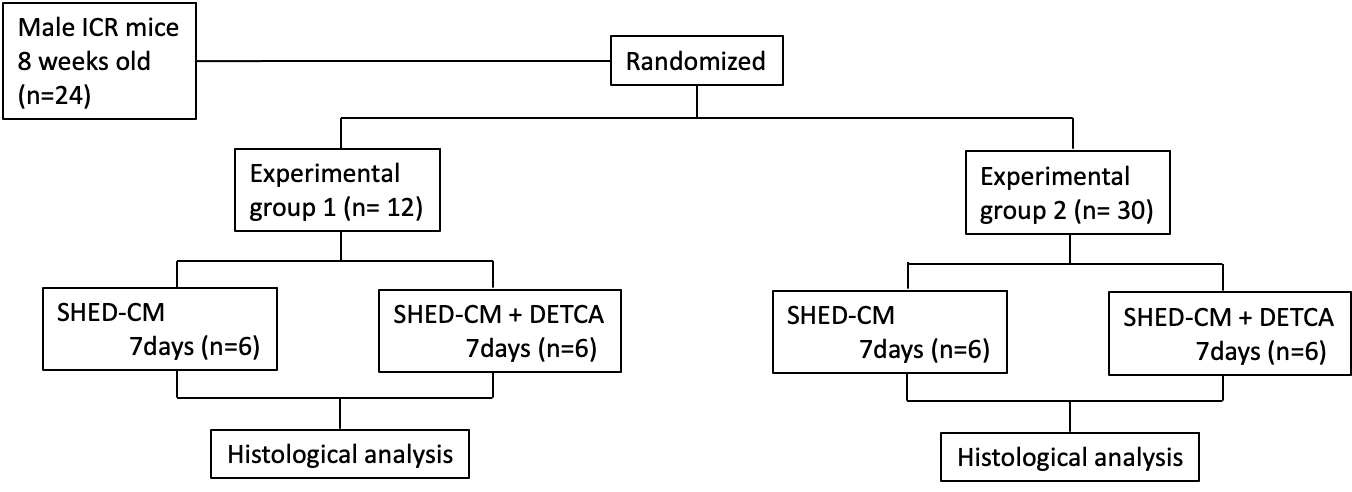
**(C)

**Supplement Figure 1. Overview of experimental design and workflow.** (A) SHED-CM, Fibro-CM or DMEM injection Experimental Design for histological, real time PCR, Oviblot analysis and SOD activity analysis. (B) SHED-CM and Fibro-CM injection Experimental Design for real time PCR of antioxidative genes. (C) SHED-CM and Fibro-CM injection Experimental Design for SOD inhibition analysis.

Full unedited blots for Fig. 3A.


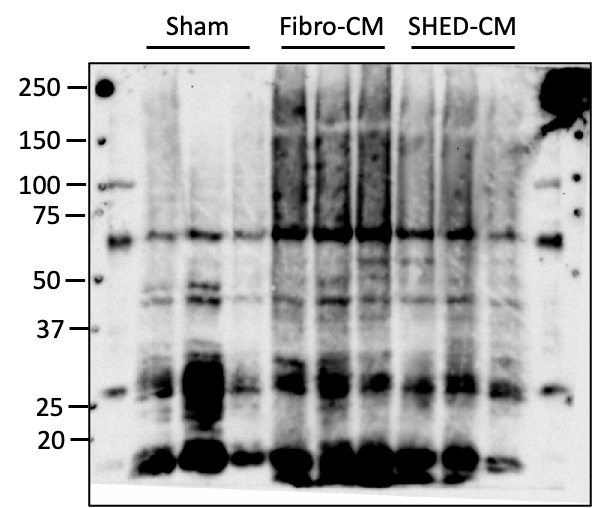


**Supplement Figure 2. Images in Fig. 3A were indicated by red rectangles.**

Supplemental Table. 1

| Gene |  | Primer sequence |  |
| --- | --- | --- | --- |
| mouse GAPDH | Forward | AACTTTGGCATTGTGGAAGG | |
| mouse GAPDH | Reverse | GGATGCAGGGATGATGTTCT | |
| mouse AQP5 | Forward | TACTTCTACTTGCTTTTCCCCTCCT | |
| mouse AQP5 | Reverse | AGGAGGGGAAAAGCAAGTAGAAGTA | |
| mouse AMY1A | Forward | GAAATGGCCGTGTGACAGAA | |
| mouse AMY1A | Reverse | AAGGCATCAAACCCCAACC | |
| mouse E-cad | Forward | CTGACCAGCAGTTCGTTGTTG | |
| mouse E-cad | Reverse | GGGTTCCTCGTTCTCCACTCT | |
| mouse ZO-1 | Forward | AAACCCGAAACTGATGCTGTG | |
| mouse ZO-1 | Reverse | CCCTTGGAATGTATGTGGAGAGA | |
| mouse CK7 | Forward | GAACAGAAGTCAGCCAAGAGCA | |
| mouse CK7 | Reverse | GCATGTTCCGCAGTTCCA | |
| mouse CK18 | Forward | TATCCGTGTCCCGCTCTGT | |
| mouse CK18 | Reverse | TGGTCTCCTTCTCGGTCTGG | |
| mouse SOD1 | Forward | AATGGTGGTCCATGAGAAACAAG | |
| mouse SOD1 | Reverse | GCAATCCCAATCACTCCACA | |
| mouse SOD2 | Forward | TCCCAGACCTGCCTTACGA | |
| mouse SOD2 | Reverse | TCGGTGGCGTTGAGATTG | |
| mouse SOD3 | Forward | GTCCAGCTTCGACCTAGCAGA | |
| mouse SOD3 | Reverse | AGCATCCACCTCCCTTCGT | |
| mouse catalase | Forward | CATAGCCAGAAGAGAAACCCACA | |
| mouse catalase | Reverse | ACAAGAAAGAAACCTGATGGAGAGA | |
| human SOD1 | Forward | GATGGTGTGGCCGATGTGT | |
| human SOD1 | Reverse | TCCAGCGTTTCCTGTCTTTGT | |
| human SOD2 | Forward | GGTTGGCTTGGTTTCAATAAGG | |
| human SOD2 | Reverse | CACACATCAATCCCCAGCA | |
| human SOD3 | Forward | GAGATCTGGCAGGAGGTCAT | |
| human SOD3 | Reverse | GAACTGGTGCACGTGGATG | |
| human catalase | Forward | GCCTGGGACCCAATTATCTT | |
| human catalase | Reverse | GAATCTCCGCACTTCTCCAG | |
| human NQO-1 | Forward | CTGGCCCATTCAGAGAAGAC | |
| human NQO-1 | Reverse | GTCTGCAGCTTCCAGCTTCT | |
| human HO-1 | Forward | CTTCTTCACCTTCCCCAACA | |
| human HO-1 | Reverse | GCTCTGGTCCTTGGTGTCAT | |
| human GPX-1 | Forward | ACACCGAGATGAACGATCTG | |
| human GPX-1 | Reverse | ATGTACTTGGGGTCGGTCAT | |
| human NRF-2 | Forward | ACATCCTTTGGAGGCAAGAC | |
| human NRF-2 | Reverse | GGGAATGTCTCTGCCAAAAG | |
| human GAPDH | Forward | TGAAGGTCGGAGTCAACGGATT | |
| human GAPDH | Reverse | CATGTGGGCCATGAGGTCCACCAC | |
